# A Novel Low-Cost Compact High-Performance Flower-Shaped Radiator Design for Modern Smartphone Applications

**DOI:** 10.3390/mi14020463

**Published:** 2023-02-16

**Authors:** Zaheer Ahmed Dayo, Muhammad Aamir, Ziaur Rahman, Imran A. Khoso, Mir Muhammad Lodro, Shoaib Ahmed Dayo, Permanand Soothar, Muhammad Salman Pathan, Ahmed Jamal Abdullah Al-Gburi, Aftab Ahmed Memon, Bhawani Shankar Chowdhry

**Affiliations:** 1 College of Computer Science, Huanggang Normal University (HGNU), Huanggang 438000, China; 2 College of Electronic and Information Engineering, Nanjing University of Aeronautics and Astronautics (NUAA), Nanjing 211106, China; 3 Wolfson School of Mechanical, Electrical and Manufacturing Engineering, Loughborough University, Loughborough LE11 3TU, UK; 4 School of Electrical and Electronic Engineering, University of Nottingham, Nottingham NG7 2RD, UK; 5 Department of Industrial Engineering, Universita Degli Studi Di Salerno (University of Salerno), Via Giovanni Paolo II, 132-84084 Fisciano, SA, Italy; 6 School of Electronic and Optical Engineering, Nanjing University of Science and Technology (NJUST), Nanjing 210094, China; 7 Department of Telecommunication and Electronics Engineering, Institute of Information and Communication Technologies (IICT), Mehran University of Engineering and Technology (MUET), Jamshoro 76062, Pakistan; 8 Department of Computer Science, National University of Ireland, Maynooth, W23 F2H6 Kildare, Ireland; 9 Microwave Research Group (MRG), Centre for Telecommunication Research & Innovation (CeTRI), Fakulti Kejuruteraan Elektronik dan Kejuruteraan Komputer (FKEKK), Universiti Teknikal Malaysia Melaka (UTeM), Hang Tuah Jaya, Durian Tunggal 76100, Malaysia

**Keywords:** low-cost, compact, high-performance, flower-shaped radiator (FSR), modern smartphone wireless applications

## Abstract

This manuscript examines the design principle and real-world validation of a new miniaturized high-performance flower-shaped radiator (FSR). The antenna prototype consists of an ultracompact square metallic patch of 0.116λ_0_ × 0.116λ_0_ (λ_0_ is the free space wavelength at 3.67 GHz), a rectangular microstrip feed network, and a partial metal ground plane. A novel, effective, and efficient approach based on open circuit loaded stubs is employed to achieve the antenna’s optimal performance features. Rectangular, triangular, and circular disc stubs were added to the simple structure of the square radiator, and hence, the FSR configuration was formed. The proposed antenna was imprinted on a low-cost F4B laminate with low profile thickness of 0.018λ_0_, relative permittivity ε_r_ = 2.55, and dielectric loss tangent δ = 0.0018. The designed radiator has an overall small size of 0.256λ_0_ × 0.354λ_0_. The parameter study of multiple variables and their influence on the performance results has been extensively studied. Moreover, the impact of different substrate materials, impedance bandwidths, resonance tuning, and impedance matching has also been analyzed. The proposed antenna model has been designed, simulated, and fabricated. The designed antenna exhibits a wide bandwidth of 5.33 GHz ranging from 3.67 to 9.0 GHz at 10 dB return loss, which resulted in an 83.6% fractional impedance bandwidth; a maximum gain of 7.3 dBi at 8.625 GHz; optimal radiation efficiency of 89% at 4.5 GHz; strong intensity current flow across the radiator; and stable monopole-like far-field radiation patterns. Finally, a comparison between the scientific results and newly published research has been provided. The antenna’s high-performance simulated and measured results are in a good agreement; hence, they make the proposed antenna an excellent choice for modern smartphones’ connectivity with the sub-6 GHz frequency spectrum of modern fifth-generation (5G) mobile communication application.

## 1. Introduction

The tremendous growth in antenna design has boosted the wireless communication industry. More recently, wireless communication systems such as smartphones, laptops, tablets, modern electronic devices, etc., have begun to shrink, and the size of antennas is becoming smaller [1,2]. Furthermore, the multifaceted components used in wireless communication systems, for example, integrated circuits (ICs), processors, cameras, speakers, batteries, etc., are making wireless systems bigger and are causing them to occupy a huge amount of space. Therefore, with the little available space in wireless communication systems, researchers are trying to design compact, high-performance antennas. Recently, the extensive use of compact, low-profile, and conformal antennas for modern 5G smartphones has been picked up upon. The modern technology covers fifth-generation (5G) cellular personal communication systems (PCS), global positioning systems (GPSs), satellite navigation, and many more practical wireless applications.

In advanced wireless communication systems, compact, low-profile designs with differently shaped radiators and metallic ground planes are being used [3,4]. The antennas exhibit high-performance features and may satisfy the plethora of modern communication system requirements using as little space as possible. Today, the requirement for planar, compact, low-cost, high-performance radiators on the market is growing very fast. Antennas that are economical, small in size, and lightweight and that have enhanced characteristics fulfill the market demand [5]. Moreover, it is very important to select a suitable type of antenna, which has the above advantages and may exhibit the characteristics of high performance. Therefore, how to design a miniaturized antenna with the best performance features is still a challenging task for the radio frequency (RF) component design community.

In the past decades, many researchers have worked on planar monopole antennas (PMAs). Different types and techniques, i.e., cutting slots, slits, and loaded stubs, have been reported in the literature to enhance impedance BW and gain and radiation characteristics [6,7]. These topologies have been implemented on different types of PMAs. Moreover, the reduction in the size of the antenna is also a big challenge for current researchers. However, due to the small size of antennas, the performance of the antennas reported in the literature is not satisfactory. Therefore, with this aim and to solve the identified research problem, compact, low-cost PMAs were designed to achieve outstanding performance in comparison to the previous literature. Recently, PMAs with diverse shapes have been identified as being ideal for contemporary wireless systems. Moreover, various antenna types have been reported in the literature. A narrower bandwidth (BW), excellent gain, radiation efficiency, and stable radiation performance with large dimensions are the main concerns for PMAs [8,9,10]. Therefore, antenna designers are working to improve the aforementioned features of PMAs. Moreover, several antenna types with multiple specifications and applications have been reported in the literature. The reported antenna design still faces a lot of challenges, for example, sensible antenna behavior, narrow BW, low efficiency, stable monopole-like patterns, acceptable gain with comparatively larger sizes, and so on.

### 1.1. Related Work

Many researchers have proposed different types of planar antennas (PMAs) with optimal performance features. The literature reports on broadband PMAs of differently shaped radiation antennas and modified ground planes. Moreover, different techniques, namely, metamaterial resonators, slots of different shapes, tunable stubs, and the selection of feed lines, have been focused upon. These techniques have been used for the improvement of impedance BW and peak realized gain. An existing study proposed an ultrahigh-frequency (UHF) miniature antenna using a high impedance surface (HIS), as reported in [11]. The antenna exhibited high gain and a reasonable BW with a larger size. Another study focused on the radiation performance improvement of a slot antenna using an artificial magnetic conductor (AMC) approach [12]. The design of mashed patch antennas for the enhancement of bandwidth (BW) has been reported [13]. The authors utilized a proximity coupling concept in the designed antennas. Small antennas integrated with metasurfaces were proposed for the improvement of BW and gain and analyzed with characteristic mode analysis (CMA) [14,15]. Similarly, the authors designed a differential-fed patch broadband antenna under TM10 and TM30 operational modes [16]. The authors analyzed and achieved a 13% increment in BW, a 7.0 dBi gain, and 85% radiation efficiency. Another study focused on several parasitic patches and shorting vias for enhancing BW, as reported in [17]. The authors attained fractional impedance BWs of 13.8% and 17.4%. A miniature broadband antenna for fifth-generation (5G) wireless communication applications has been presented in [18]. The authors achieved a fractional impedance bandwidth (FIBW) of 58.3% and stable gain performance of 5.0 dBi in the operational band. An elliptical ring ultrawideband (UWB) antenna for radio detection and ranging (RADAR) imaging applications was designed [19]. Overall, the antenna possessed larger dimensions and exhibited a reasonable gain performance of 4.0 dBi. A flower-shaped antenna with an enhanced BW for portable wireless devices is demonstrated in ref. [20]. The proposed antenna was embedded on 54 × 64 × 1.6 mm^3^ laminate and exhibited an FIBW of 49% and a maximum gain of 3.4 dBi.

Recently, two compact broadband patch antennas were designed with inverted L- and T-shaped strips [21]. The reported work achieved (12.46; 15) % FIBW, (6.6; 6.25) dBi gain, and (84; 78) % radiation efficiency in the operational band. A hybrid mode dipole antenna for sub-6 GHz wireless applications is reported in ref. [22]. The designed antenna achieved 67.5% FIBW and a maximum gain of 8.4 dBi with design complexity and a larger size. A compact broadband and high gain differential-fed antenna under tri-mode resonance has been proposed [23]. The antenna achieved 55% FIBW and a peak gain >10 dBi. The designed structure has a larger size; the design complexity and radiation efficiency of the antenna were not analyzed and reported. Another magneto-electric dipole antenna driven with a differential-fed network has been reported [24]. The antenna exhibited 62% FIBW and the gain variation was observed from 6.6 to 9.6 dBi in the operable frequency span. However, the suggested antenna is imprinted on a larger sized laminate and suffers from design complexity. A low-profile, high-gain antenna integrated with metasurfaces was designed in ref. [25]. The antenna attained 28.4% FIBW and a peak gain of 8.2 dBi. A small dual band antenna for sub-6 GHz of the 5G microwave spectrum is proposed in ref. [26]. The radiator has the overall size of 31 × 36 × 0.8 mm^3^ and obtained reasonable performance. Another piece of research on a low-profile antenna with enhanced performance features was presented in ref. [27]. A new antenna design with a stair-shaped metal ground plane was proposed in ref. [28]. The core focus of this research was on the BW increment, and it achieved polarization reconfigurable features. Moreover, a rectangle microstrip line feed wide slot antenna structure with fractal-shaped and rotated square-shaped slots was proposed [29,30]. The presented antennas achieved acceptable performance in terms of gain and BW. Modified geometries of the antennas based on a crescent-shaped patch, disc-shaped slots loaded on the main hexagonal-shaped slot and symmetrical triangular-shaped slots for versatile wireless applications have been designed in refs. [31,32]. The reported antennas achieved a wideband and multiresonant behavior with stable radiation performance. A flower-shaped radiator coplanar waveguide (CPW)-fed structure for wideband applications was reported [33]. The authors performed a polyline function operation on a simple, circular-shaped patch to form the new shape of the radiator. An ultracompact broadband monopole antenna with an elliptical radiator and trapezoidal ground plane is proposed in ref. [34]. The authors achieved good performance by utilizing a mixed approach.

A modified structure of a patch antenna with the triangular-shaped slot and stepped cut on the radiator was demonstrated in ref. [35]. The authors attained the broadband features with substantial gain performance. A compact bow-tie antenna of 122 × 56 mm^2^ in size excited with a tapered feed structure has been proposed in ref. [36]. The authors achieved broadband features and a gain performance of around 6.5 dBi with modified rectangular extensions to the bow-tie arm structure. An hourglass and an octagonal Sierpiński-shaped monopole antenna feed with an asymmetrical CPW and a microstrip feedline was reported in refs. [37,38]. The antennas exhibited good performance regarding their wideband, gain, efficiency, and radiation patterns. A miniature cross-shaped antenna with a modified ground plane was presented in ref. [39]. The designed antenna exhibited wide circular polarization features and stable pattern performance. All of these antennas have a complex geometry and a large size. Furthermore, a broadband antenna for the global navigation satellite systems (GNSSs) and wireless fidelity (Wi-Fi) wireless applications has been proposed [40]. The antenna in the study exhibited substantial performance features and antenna gain; radiation efficiency was not focused upon in the study. A conformal wideband antenna with loaded meandered arms for wireless capsule endoscopy was designed in ref. [41]. The authors only presented the design simulation results and the results were not experimentally verified. A superstrate resonant cavity antenna with a via hole-based patch was presented in ref. [42]. The authors obtained a high gain 15.8 dBi and a reasonable FIBW of 10.7%. The reported antenna faced the design complexity, and its efficiency was not analyzed. Similarly, multiple designs of E-shaped reconfigurable patch antennas using RF switches were reported in ref. [43]. However, the efficiency and gain of the antennas were not analyzed, and the authors provided only the simulation study. A hexagonal probe-fed radiator was presented in [44]. The reported antenna occupied a larger space and achieved a wide impedance BW of 8.3 GHz. A narrow frame and a hybrid mode with slotted antennas for mobile communication and sub-6 GHz wireless applications presented in refs. [45,46]. The antennas exhibited optimal performance features with larger dimensions. Differently shaped antennas were reported [47,48]. The authors designed bow-tie-shaped and bio-inspired antennas and analyzed the different substrate materials’ impact on the designed dimensions and achieved a wide impedance BW.

### 1.2. Key Contributions

In this article, a new miniaturized low-cost, high-performance, flower-shaped radiator (FSR) for modern smartphones was simulated and designed. The antenna structure consisted of a very small square metallic patch of 9.5 × 9.5 mm^2^, a rectangular microstrip feed structure, and a metal ground plane. A novel, effective, and efficient approach based on an open circuit loaded rectangular, triangular, and circular disc stub was employed to achieve the antenna’s optimal performance features. The proposed antenna is engraved on a low-cost F4B substrate material with overall compact dimensions of 21 × 29 mm^2^. Furthermore, the parameter study related to the multiple variables and their impact on the performance results was extensively studied. The impact of different substrate materials, impedance BWs, resonance tuning, and impedance matching was also analyzed. The proposed antenna model was designed, simulated, and fabricated. The designed antenna exhibited a broad BW of 5.33 GHz in the working frequency ranging from 3.67–9.0 GHz at 10 dB return loss [49]; a maximum gain of 7.3 dBi at 8.625 GHz; optimal efficiency of 89% at 4.5 GHz; strong intensity current flow through the radiator; and stable, monopole-like far-field radiation patterns. Finally, a comparison between the scientific results and newly published research has been provided. The antenna’s high-performance simulated and measured results are in good agreement and hence make the proposed antenna an excellent choice for modern smartphones’ connectivity with the sub-6 GHz frequency spectrum of modern fifth-generation (5G) wireless communication systems.

The core contributions of this manuscript are as follows:

🏶 It examines design principles and real-world validation of a new flower-shaped radiator with a compact size of 21 × 29 mm^2^.
🏶 A novel, effective, and efficient approach based on open circuit loaded stubs is employed to achieve the antenna’s optimal performance features.
🏶 Frequency tuning and impedance matching can be attained with the flexible usage of variables.
🏶 The designed antenna exhibits high-performance features including a broad bandwidth of 5.33 GHz ranging from 3.67–9.0 GHz at 10 dB return loss; a maximum gain of 7.3 dBi at 8.625 GHz; optimal radiation efficiency of 89% at 4.5 GHz; strong intensity current flow across the radiator; and stable monopole-like far-field radiation patterns. Finally, a comparison between the scientific results with newly published research has been provided.
🏶 The proposed radiator is a very suitable candidate for modern smartphones’ connectivity with the sub-6 GHz frequency spectrum of modern fifth-generation (5G) mobile communication applications.

### 1.3. Manuscript Structure

The rest of the paper is structured as follows: Section 2 explains the design methodology of the proposed radiator. Section 3 analyzes the simulation results. Section 4 introduces the experimentally validated results of return loss, efficiency, gain, and far-field radiation patterns, and recently published review literature results are given in Section 5. Finally, the concluding remarks are briefly explained in Section 6.

## 2. Proposed Antenna Design Methodology

**Figure 1:**
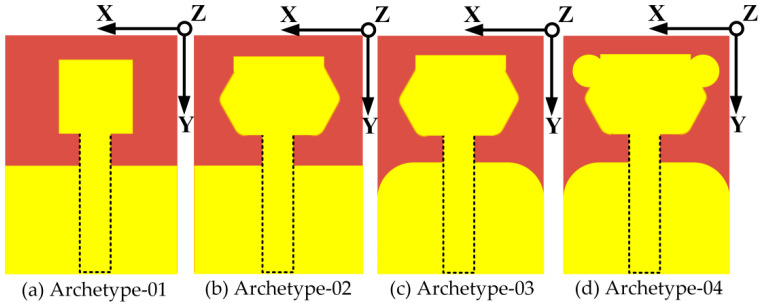

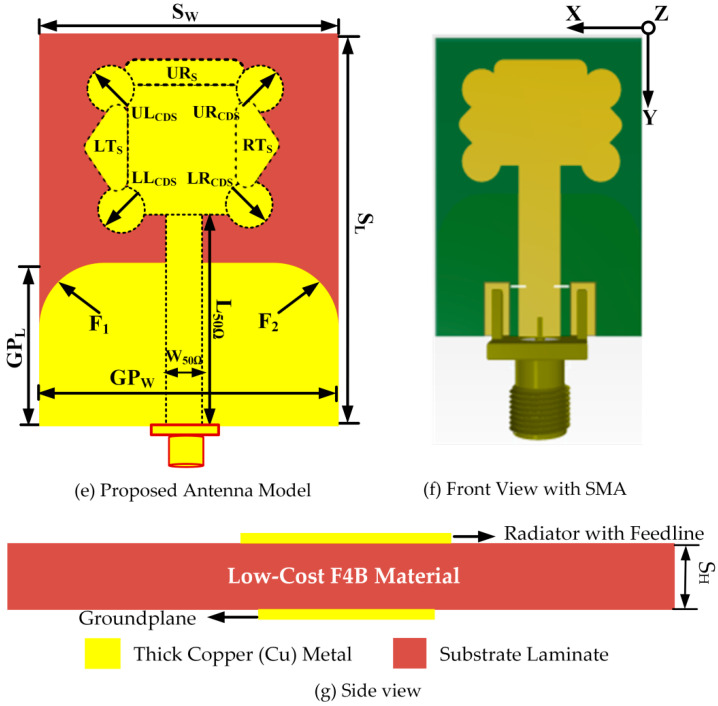
Diagram of the process of the antenna: (**a**) feeding the square patch with a partial ground plane, (**b**) a modified patch loaded with a stub, (**c**) a modified patch with a chamfered partial ground plane, (**d**) a loaded patch with a disc-shaped stub, (**e**) a top and back view of the proposed antenna, and (**f**) a 3D model of the designed antenna and (**g**) side view of the proposed antenna.

The antenna development phases are shown in Figure 1a–d. Initially, the simple antenna structure is comprised different elements such as a compact square patch of 9.5 × 9.5 mm^2^, a rectangular feed line of 16.6 × 4.23 mm^2^, and a partial ground plane (PGP) with a compact size of 14 × 21 mm^2^. The antenna elements are etched onto a low-cost, 1.5 mm thick F4B substrate with a relative permittivity of 2.55 and dielectric loss tangent of 0.0018. The elements of the antenna are made of copper with a value of 0.035 mm. Furthermore, as portrayed in Figure 1b, an evolved antenna structure is formed by modifying the metallic compact square patch. Firstly, two rectangular stubs are loaded on the left and right sides of a compact metal square patch with a miniaturized size of 4.0 × 8.15 mm^2^. Then, polyline operations are performed on both of the sides of the loaded rectangular stubs to form triangular-shaped stubs. As elucidated in Figure 1c, the third step in the antenna design process is to perform a 5.0 mm fillet operation on the upper edges of the metal part of the ground plane. Modification in the antenna’s design leads to the enhancement of the impedance bandwidth and the realization of proper impedance matching. Moreover, the fourth step in the antenna design is realized by having compact 2.0 mm loading disc stubs at the upper left and right edge of the improved metal patch as shown in Figure 1d.

The proposed antenna design is formed by more than two disc-shaped stubs, and the compact size of 2.0 mm is loaded on the lower edges of the modified metal patch. It was noticed that by loading several stubs on a simple square patch, the antenna can have a novel, flower-shaped patch. The dimensions of the antenna’s key elements, such as the feedline, metal patch, dielectric laminate, and loading stubs, have a great influence on the overall performance of the antenna. The antenna was designed and simulated by using a three-dimensional (3D) electromagnetic simulation solver (HFSS). The top, bottom, and side view of the proposed antenna are portrayed in Figure 1e,g. The proposed antenna was carefully designed in the industrial software tool of the 3D Altium designer (AD) printed circuit board (PCB), as shown in Figure 1f. The optimized parameters are listed in Table 1.

**Figure 2:**
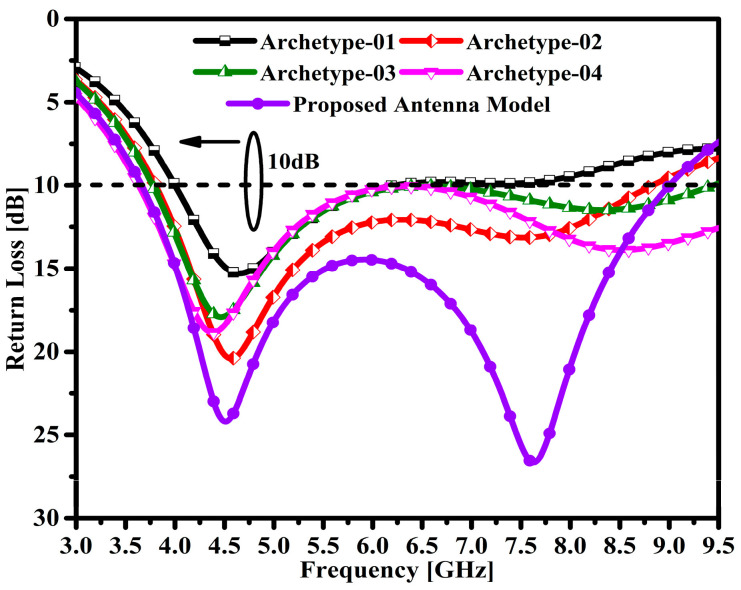
Corresponding return loss |S_11_| in the antenna design process.

Figure 2 shows the return loss performance of the evolved antenna models over the entire frequency range. The first designed antenna model achieved a bandwidth (BW) of 2.19 GHz with a return loss of 10 dB, ranging from 4.0–6.19 GHz, which can be observed in the graph.

The second prototype antenna achieves an impedance bandwidth of 2.46 GHz with a return loss of 10 dB, ranging from 3.78–6.24 GHz. Similarly, the third prototype antenna achieves an impedance bandwidth of 2.65 GHz with a return loss of 10 dB, ranging from 3.63–6.28 GHz. Moreover, the fourth prototype antenna achieves a broader impedance bandwidth of 5.1 GHz with a return loss of 10 dB, ranging from 3.7–8.8 GHz. The proposed antenna model achieves a wide impedance bandwidth of 5.33 GHz with a return loss of 10 dB, ranging from 3.67–9.0 GHz. Moreover, under the return losses of 24.18 dB and 26.6 dB, two resonances are observed at 4.52 GHz and 7.64 GHz, respectively.

## 3. Simulation Results and Analysis

The parameter study of the important variables that affect the impedance BW, the matching and tuning performance of the proposed antenna, is explained in this section.

### 3.1. The Impact of the Ground Plane and 50 Ω Feedline Dimensions

**Figure 3:**
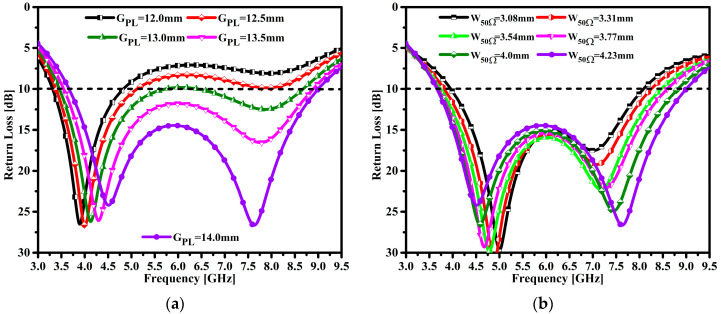
Influence of return loss on operating frequency, (**a**) length of the ground plane, and (**b**) the width of the 50 Ω feedline.

The size and length of the ground plane and the width of the patch (WoP) have an influence on the proposed antenna impedance matching performance and resonance tuning features. Figure 3a shows the influence of the length of the ground plane (G_PL_) over the frequency span. It can be analyzed that when the G_PL_ is 14.0 mm, the frequency tuning and impedance are perfectly matched. Similarly, it can be observed that when the values of G_PL_ are at the initial stage, the impedance performance and tuning at the first resonance point do not match. Figure 3b elucidates the influence of the 50 Ω feeder width on the frequency span. It can be seen that at a maximum value of feedline width (W_50Ω_), the perfect impedance matching and broad impedance bandwidth are achieved. In addition, lower values of feedline width strongly affect the impedance bandwidth. Likewise, the frequency tuning of resonances is observed at the lower value of W_50Ω_. From the above analysis, it can be concluded that the G_PL_ and W_50Ω_ have a great influence on the impedance matching, BW, and resonance tuning in the operable frequency span.

### 3.2. The Impact of Substrate Height and Material

**Figure 4:**
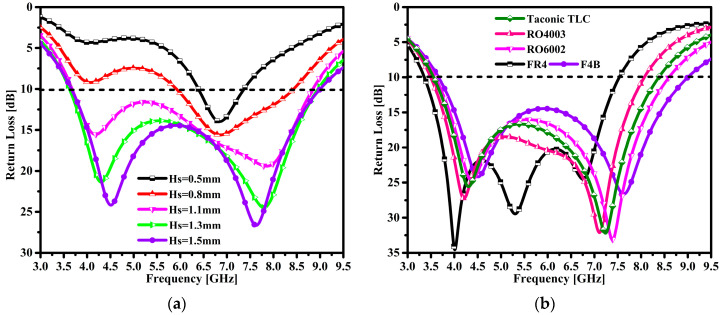
Parametric study of defined variables: (**a**) impact of substrate height and (**b**) impact of different substrate materials.

The proposed antenna substrate thickness influence is shown in Figure 4a. At this time, the antenna achieves a narrow impedance bandwidth of 0.97 GHz at the lowest value of 0.5 mm and with the operating frequency band ranging from 6.39 to 7.36 GHz. One can see single resonance is observed at 6.86 GHz with a return loss peak at 13.97 dB. Furthermore, the thickness of the substrate was increased to obtain a broader impedance bandwidth. It can be observed that at 0.8 mm thickness, the antenna bandwidth is slightly improved. The antenna achieved an impedance BW of 2.51 GHz, ranging from 5.92 to 8.43 GHz. The single resonance was obtained at 6.86 GHz with a peak return loss of 15.6 dB. The required target of the broader impedance BW had not been achieved yet. Furthermore, when the substrate was 1.1 mm thick, the antenna achieved a broader impedance BW of 5.17 GHz ranging from 3.67 to 8.84 GHz. Two resonances were observed at 4.19 GHz and 7.8 GHz with peak return losses of 15.5 dB and 19.4 dB, respectively. When the thickness of the substrate was increased to 1.3 mm, it can be seen that the proposed antenna exhibited an impedance BW of 5.33 GHz in the range of 3.67 to 9.0 GHz with a return loss of 10 dB. In addition, at the two resonance points of 4.31 GHz and 7.8 GHz, the peak return losses were 21.3 dB and 24.4 dB, respectively. Finally, the optimal value of the substrate thickness was chosen as 1.5 mm, and the antenna achieved a broader impedance BW of 5.32 GHz in the frequency range of 3.63–8.95 GHz. Additionally, two resonances can be observed at 4.52 GHz and 7.62 GHz with peak return losses of 24 dB and 28.2 dB, respectively. From the above analyzed results, it can be observed that the thickness of the substrate plays an important role in obtaining the broad impedance BW of the proposed antenna.

The substrate material of the proposed antenna was carefully selected to achieve optimal results. Figure 4b provides the performance of different substrate materials across the operable frequency span. The influence of five different substrate materials such as Taconic TLC, RO4003, RO6002, FR4, and F4B on the impedance bandwidth performance has been extensively analyzed. Initially, the Taconic TLC laminate with a relative permittivity of 3.2 and a dielectric loss tangent of 0.03 was chosen to analyze the return loss performance of the proposed antenna across the operable frequency span. It can be seen that the antenna achieved a 4.81 GHz impedance BW in the 3.56–8.37 GHz range with a return loss of 10 dB. Another substrate material, namely, Rogers RO4003 with a relative permittivity of 3.55 and a dielectric loss tangent of 0.0027 was selected. The antenna achieved a 4.5 GHz impedance BW ranging from 3.5 to 8.0 GHz under 10 dB return loss. Likewise, the Rogers RO6002 substrate material with a relative permittivity of 2.94 and a dielectric loss tangent value of 0.0012 was chosen to obtain a broad impedance BW. The antenna obtained an impedance BW of 4.99 GHz ranging from 3.6 to 8.59 GHz with a return loss of 10 dB. The low-cost FR4 epoxy material with a relative permittivity of 4.4 and a dielectric loss tangent value of 0.02 was selected to achieve a broader impedance BW. With the selected material, the antenna achieved a broader impedance BW of 4.17 GHz ranging from 3.36 to 7.53 GHz with a return loss of 10 dB. Finally, a low-cost F4B material was selected to achieve a broader impedance BW for the proposed antenna. The proposed antenna achieved a broader impedance BW of 5.32 GHz ranging from 3.63 to 8.95 GHz with a return loss of 10 dB.

### 3.3. The Intensity of the Current across the Radiator

**Figure 5:**
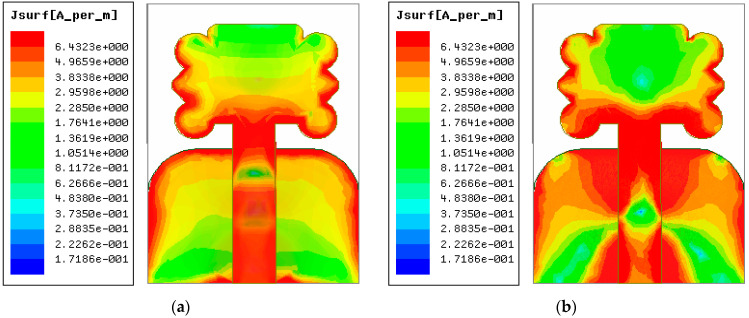
Current intensity of the antenna surface at different resonant frequencies: (**a**) at 4.52 GHz and (**b**) at 7.64 GHz.

Figure 5 portrays the current distribution on the flower-shaped radiator surface corresponding to two resonance points at 4.52 GHz and 7.64 GHz. It can be seen that the proposed antenna exhibits a strong distribution of current on the feedline, the edges of patch, and the edges of the partial ground plane. Moreover, it can also be seen that the proposed antenna current in the proposed operating band is very strong, so it is very suitable for the lower sub 6 GHz band of the fifth-generation (5G) spectrum.

## 4. Experimental Verified Results

The simulation and measurement results of return loss (|S_11_|), peak realized gain, radiation efficiency, and the performance of the proposed antenna radiation pattern are included in this section.

### 4.1. Return Loss (|S_11_|)

**Figure 6:**
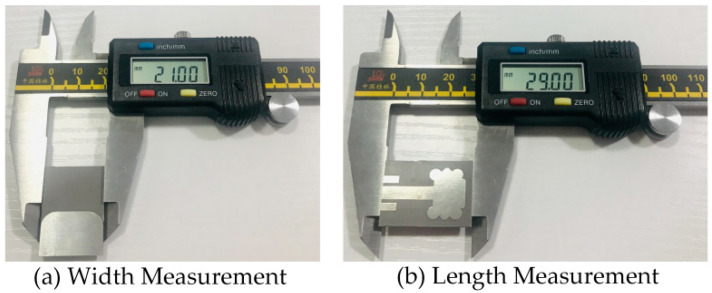
Manufactured antenna prototype: (**a**) back view and (**b**) front view.

**Figure 7:**
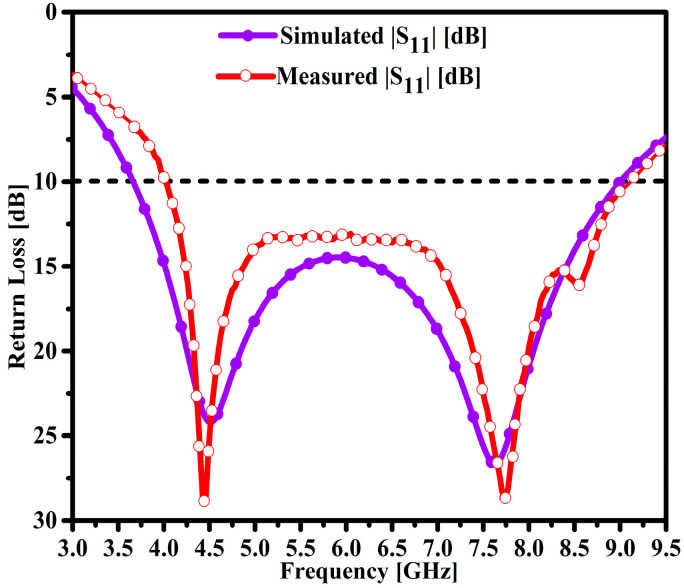
Simulated and tested |S_11_| performance of the proposed antenna.

The fabricated model of the radiating structure is shown in Figure 6a,b. The antenna model is connected to one port of the calibrated Agilent vector network analyzer (VNA) model no. PNA5230C. The simulation and measurement results of the proposed and fabricated antenna sample were compared and are elucidated in Figure 7. It can be seen that the designed antenna model achieved an impedance BW of 3.67–9.0 GHz, which constitutes a wider impedance BW of 5.33 GHz. Similarly, two resonances were observed at 4.52 GHz and 7.64 GHz and the maximum return losses of 24.18 dB and 26.6 dB. Moreover, the fabricated antenna sample exhibits similar responses to the simulated results. However, small discrepancies in the measurement results were observed due to substrate losses and imperfect soldering of an SMA connected to the proposed antenna feedline.

### 4.2. Performance Peak Gain and Efficiency (η)

**Figure 8:**
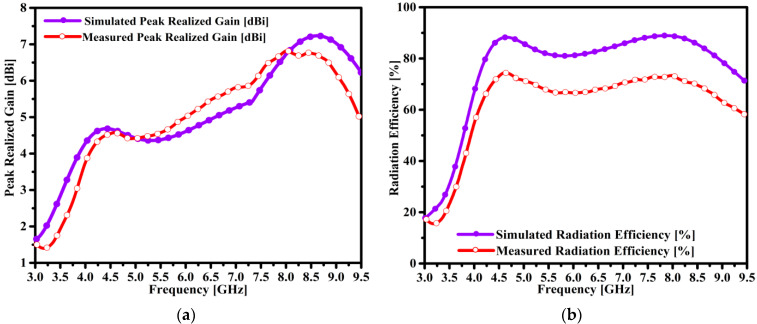
Comparing the simulation and measurement results of the antenna, (**a**) the peak realized gain, and (**b**) the radiation efficiency.

The peak gain performance of the fabricated prototype is measured by two antenna methods with a priori knowledge of the gain values. Figure 8a,b shows the fabricated sample and the ridge gap horn antennas’ placement inside the chamber room. Figure 8a compares the simulated and tested results of the peak gain of the proposed antenna. The designed antenna exhibits a reasonable average gain of 4.1 dBi at 3.91 GHz. In addition, gain performances of 4.3 dBi and 6.125 dBi were observed at 4.52 GHz and 7.64 GHz, respectively. It can be observed that the gain of the antenna was increased monotonically from 4.15 to 7.0 dBi in the range of 3.9–8.25 GHz. Moreover, the simulation results show that the broadside peak gain values were 7.3 dBi to 7.0 dBi at 8.625 GHz and 8.25 GHz, respectively. The measurement results of the antenna sample coincided with the simulation results. Moreover, reasonable discrepancies of around 0.5 dB were observed in the measurement results. This loss is attributed to the measurement of the fabricated antenna sample and also may be due to the lossy substrate and real-time environment losses inside the anechoic chamber room.

The radiation efficiency of the proposed antenna was extracted by an efficient, accurate, and simple gain directivity method. From Figure 8b, it can be seen that the antenna exhibited the maximum radiation efficiency of 89% at 4.5 GHz. Likewise, a reasonable level of radiation efficiency such as 39% and 50% at 3.67 GHz and 3.75 GHz, respectively, was observed. The antenna obtained excellent efficiency performance of around 86.5% and 87% at the frequencies of 4.52 GHz and 7.64 GHz, respectively. Moreover, the measurement results of radiation efficiency show a loss of almost 15% as compared to the simulation results. These losses are mainly due to the approach employed for the radiation efficiency measurement.

### 4.3. Far-Field Radiation Performance

**Figure 9:**
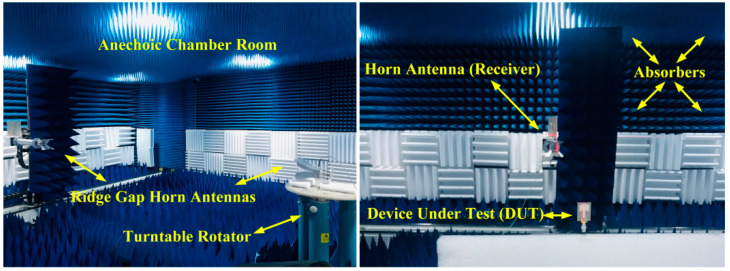
Placement of antennas in the anechoic chamber ridge gap horn antennas and DUT.

**Figure 10:**
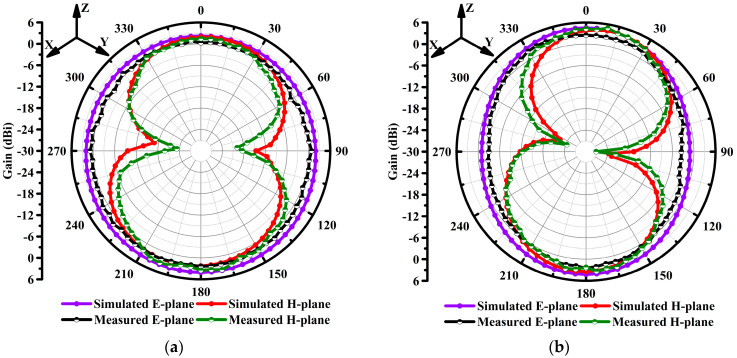
Radiation pattern performance (Φ = 0°) and (Φ = 90°) at different resonant frequencies: (**a**) at 4.52 GHz and (**b**) at 7.64 GHz.

The fabricated antenna sample was measured inside an anechoic chamber room. The antenna under test (AUT) was placed on a turntable and rotated at 360° as shown in Figure 9. In Figure 10a,b, the simulation and measurement results of the far-field two-dimensional (2D) radiation pattern of the antenna obtained on the standard planes are compared and illustrated.

The proposed antenna exhibits a perfect monopole-like radiation pattern at both resonances. Figure 10a is the radiation pattern performance at the 4.52 GHz resonant point and the proposed antenna radiates omnidirectionally in the E and H planes. The measurement results at the resonant frequency of 4.52 GHz coincide with the simulation results. Nulls in the measured results are observed at around 90° and 270° in the H plane at the resonant frequency of 4.52 GHz. Moreover, the 2D radiation patterns at the second resonant frequency of 7.64 GHz are shown in Figure 10b. The proposed antenna exhibits a perfect omnidirectional radiation pattern in the E and H planes. Furthermore, the nulls are obtained in the H plane at 7.64 GHz. It is noted that the radiation pattern becomes deteriorated at some points. A slight and acceptable discrepancy in the measured results is observed due to the lossy nature of the cables, the power meter sensitivity, the substrate material used, subminiature-A (SMA) connector soldering, and loss of the measuring chamber.

## 5. Performance Comparison Analysis

Table 2 demonstrates the proposed comparison of the antenna’s key features with newly published work. The proposed radiator shows clear advantages in terms of size, operable frequency, fractional impedance bandwidth (FIBW), gain, radiation efficiency, and design complexity.

## 6. Concluding Remarks

In this article, a new, low-cost, compact, high-performance, flower-shaped radiator (FSR) for modern smartphones was designed and fabricated. A novel method comprising open circuit loaded stubs was employed to achieve the antenna’s optimal performance features. The designed structure of the antenna has the overall miniaturized dimensions of 21 × 29 mm^2^. The proposed antenna was imprinted on F4B substrate material with a thickness of 1.5 mm, ε_r_ = 2.55, and δ = 0.0018. The parameter study of the multiple variables and their impact on the impedance BW and the matching and resonance tuning results were extensively studied. Moreover, the impact of diverse laminate materials on the performance of the radiator was also analyzed. When the return loss was less than 10 dB, the working frequency band of the proposed antenna exhibited a broad impedance BW of 5.33 GHz ranging from 3.67 to 9.0 GHz, a maximum gain of 7.3 dBi at 8.625 GHz, optimal efficiency of 89% at 4.5 GHz, strong intensity current flow across the radiator, and stable monopole-like far-field radiation patterns were obtained. Hence, it is concluded that the proposed antenna’s high-performance simulated and measured results are in good agreement and hence make the proposed antenna an excellent choice for modern smartphone connectivity with the sub-6 GHz frequency spectrum of modern fifth-generation (5G) mobile communication applications.

## Figures and Tables

**Table 1:** Parameters of the proposed antenna (unit: mm).

Variables	Optimal Value	Variables	Optimal Value
S_L_	29.0	S_W_	21.0
S_H_	1.5	G_PW_	21.0
G_PL_	14.0	F_1_ = F_2_	5.0
W_50Ω_	4.23	L_50Ω_	16.6
UL_CDS_	2.0	LL_CDS_	2.0
LT_S_ = RT_S_	4.0 × 8.15	U_RS_	9.5 × 0.8
W_P_	9.5	L_P_	9.5

**Table 2:** Performance comparison between the latest published work and the proposed radiator.

Ref.	Electrical Dimensions (λ_0_) (S_W_ × S_L_ × S_H_)	FIBW (%)	Gain (dBi)	η (%)	Proposed Design Attributes		
					Size	Material	Occupied Space (mm^2^)
**Our work**	**0.256λ_0_ × 0.354λ_0_ × 0.018λ_0_**	**83.6**	**7.3**	**89**	**small**	**F4B**	**609**
[18]	0.84λ_0_ × 0.68λ_0_ × 0.06λ_0_	58.3	6.2	86	large	FR4	3225.6
[11]	0.062λ_0_ × 0.179λ_0_ × 0.0163λ_0_	55.6	10.25	97	large	PVC	4235
[12]	0.958λ_0_ × 1.533λ_0_ × 0.076λ_0_	86.48	10.6	Not Given (NG)	large	FR4	4000
[14]	1.78λ_0_ × 1.78λ_0_ × 0.07λ_0_	24.4	7.8	>88	small	RO4003C	9409
[15]	0.46λ_0_ × 0.46λ_0_ × 0.06λ_0_	31	14.1	NG	large	RO4003C	9409
[21]	0.67λ_0_ × 0.67λ_0_ × 0.03λ_0_	15	6.25	78	small	F4B	1600
[28]	0.68λ_0_ × 0.68λ_0_ × 0.0068λ_0_	6.1	2.9	NG	large	FR4	6400
[17]	0.72λ_0_ × 0.66λ_0_ × 0.02λ_0_	17.4	4.0	NG	small	FR4	1404
[13]	0.247λ_0_ × 0.247λ_0_ × 0.013λ_0_	2.65	>5.0	>65	small	RO4003C	900
[27]	2.53λ_0_ × 1.26λ_0_ × 0.029λ_0_	12	6.5	90	large	F4B	25,000
[26]	0.3λ_0_ × 0.17λ_0_ × 0.009λ_0_	60.6	7.17	80	small	FR4	1116
[22]	0.90λ_0_ × 0.78λ_0_ × 0.13λ_0_	67.5	8.4	67.5	large	FR4	16,900
[23]	1.05λ_0_ × 1.36λ_0_ × 0.06λ_0_	55	7.3	NG	large	RO5008	78,400
[24]	1.3λ_0_ × 1.3λ_0_ × 0.24λ_0_	68	10.3	NG	large	PEC	67,600
[25]	1.3λ_0_ × 1.3λ_0_ × 0.06λ_0_	28.4	8.2	95	large	NG	6084
[20]	0.42λ_0_ × 0.36λ_0_ × 0.011λ_0_	49	3.4	NG	large	FR4	3465
[19]	0.53λ_0_ × 0.66λ_0_ × 0.01λ_0_	111	4.1	NG	large	FR4	8000

## Data Availability

Not applicable.

## References

[1] Tong X., Jiang Z.H., Yu C., Wu F., Xu X., Hong W. (2021). Low Profile, Broadband, Dual-Linearly Polarized, and Wide-Angle Millimeter Wave Antenna Arrays for Ka-Band 5G Applications. IEEE Antennas Wirel. Propag. Lett..

[2] Cai Z., Weng Z., Qi Y., Fan J., Zhuang W. (2021). A High-Performance Standard Dipole Antenna Suitable for Antenna Calibration. IEEE Trans. Antennas Propag..

[3] Cappelletti G., Caratelli D., Cicchetti R., Simeoni M. (2011). A Low-Profile Printed Drop-Shaped Dipole Antenna for Wide-Band Wireless Applications. IEEE Trans. Antennas Propag..

[4] Balderas L.I., Reyna A., Panduro M.A., Del Rio C., Gutiérrez A.R. (2019). Low-Profile Conformal UWB Antenna for UAV Applications. IEEE Access.

[5] He D., Chen Y., Yang S. (2022). A Low-Profile Triple-Band Shared Aperture Antenna Array for 5G Base Station Applications. IEEE Trans. Antennas Propag..

[6] Le T.T., Kim Y.D., Yun T.Y. (2021). A Triple-Band Dual-Open-Ring High Gain High-Efficiency Antenna for Wearable Applications. IEEE Access.

[7] Lima de Paula I., Lemey S., Bosman D., Van den Brande Q., Caytan O., Lambrecht J., Cauwe M., Torfs G., Rogier H. (2021). Cost-Effective High-Performance Air-Filled SIW Antenna Array for the Global 5G 26 GHz and 28 GHz Bands. IEEE Antennas Wirel. Propag. Lett..

[8] Saleh W., Letestu Y., Sauleau R., Cruz E.M. (2021). Design and Measurements of a High-Performance Wideband Transmit array Antenna for D Band Communications. IEEE Antennas Wirel. Propag. Lett..

[9] Hosseini-Fahraji A., Manteghi M. (2021). Design of a Broadband High Gain End-Fed Coaxial Collinear Antenna. IEEE Antennas Wirel. Propag. Lett..

[10] Sun W., Li Y. (2021). Gain Stabilization Method for Wideband Slot-Coupled Microstrip Antenna. IEEE Trans. Antennas Propag..

[11] Mohamed-Hicho N.M., Antonino-Daviu E., Cabedo-Fabres M., Ferrando-Bataller M. (2015). A Novel Low-Profile High-Gain UHF Antenna Using High-Impedance Surfaces. IEEE Antennas Wirel. Propag. Lett..

[12] Malekpoor H., Jam S. (2016). Improved Radiation Performance of Low Profile Printed Slot Antenna Using Wideband Planar AMC Surface. IEEE Trans. Antennas Propag..

[13] Yasin T., Baktur R. (2017). Bandwidth Enhancement of Meshed Patch Antennas Through Proximity Coupling. IEEE Antennas Wirel. Propag. Lett..

[14] Liu W.E.I., Chen Z.N., Qing X., Shi J., Lin F.H. (2017). Miniaturized Wideband Metasurface Antennas. IEEE Trans. Antennas Propag..

[15] Lin F.H., Chen Z.N. (2017). Low-Profile Wideband Metasurface Antennas Using Characteristic Mode Analysis. IEEE Trans. Antennas Propag..

[16] Liu N.W., Zhu L., Choi W.W. (2017). A Differential-Fed Microstrip Patch Antenna with Bandwidth Enhancement under Operation of TM10 and TM30 Modes. IEEE Trans. Antennas Propag..

[17] Xu K.D., Xu H., Liu Y., Li J., Liu Q.H. (2018). Microstrip Patch Antennas with Multiple Parasitic Patches and Shorting Vias for Bandwidth Enhancement. IEEE Access.

[18] An W., Li Y., Fu H., Ma J., Chen W., Feng B. (2018). Low-Profile and Wideband Microstrip Antenna with Stable Gain for 5G Wireless Applications. IEEE Antennas Wirel. Propag. Lett..

[19] Darwhekar A., Dongaonkar P., Ray K.P. Design of a compact Ultrawideband Printed Elliptical Ring Monopole Antenna for Imaging Radar Application. Proceedings of the IEEE 5th International Conference for Convergence in Technology (I2CT).

[20] Gupta N., Saxena J., Bhatia K.S. (2019). Design of Wideband Flower Shaped Microstrip Patch Antenna for Portable Applications. Wirel. Pers. Commun..

[21] Boukarkar A., Satouh R. (2021). Bandwidth enhancement of compact patch antennas by loading inverted ‘L’ and ‘T’ strips. Int. J. Microw. Wirel. Technol..

[22] Huang B., Lin W., Huang J., Zhang J., Zhang G., Wu F. (2019). A patch/dipole hybrid-mode antenna for Sub-6GHz communication. Sensors.

[23] Liu N.W., Zhu L., Choi W.W., Zhang J.D. (2018). A low-profile differentially fed microstrip patch antenna with broad impedance bandwidth under triple-mode resonance. IEEE Antennas Wirel. Propag. Lett..

[24] Xue Q., Liao S.W., Xu J.H. (2013). A Differentially-Driven Dual-Polarized Magneto-Electric Dipole Antenna. IEEE Trans. Antennas Propag..

[25] Pan Y.M., Hu P., Zhang X., Zheng S.Y. (2016). A Low-Profile High-Gain and Wideband Filtering Antenna with Metasurface. IEEE Trans. Antennas Propag..

[26] Ishteyaq I., Shah Masoodi I., Muzaffar K. (2021). A compact double-band planar printed slot antenna for sub-6 GHz 5G wireless applications. Int. J. Microw. Wirel. Technol..

[27] Liu N.W., Gao S., Zhu L., Ji L.Y., Yang L., Zheng H.L. (2020). Low-profile microstrip patch antenna with simultaneous enhanced bandwidth, beam width, and cross-polarization under dual resonance. IET Microw. Antennas Propag..

[28] Yang Z.X., Yang H.C., Hong J.S., Li Y. (2014). Bandwidth enhancement of a polarization-reconfigurable patch antenna with stair-slots on the ground. IEEE Antennas Wirel. Propag. Lett..

[29] Chen W.-L., Wang G.-M., Zhang C.-X. (2009). Bandwidth Enhancement of a Microstrip-Line-Fed Printed Wide-Slot Antenna with a Fractal-Shaped Slot. IEEE Trans. Antennas Propag..

[30] Eskandari H., Naghi Azarmanesh M. (2009). Bandwidth enhancement of a printed wide-slot antenna with small slots. AEU Int. J. Electron. Commun..

[31] See C.H., Abd-Alhameed R.A., Zhou D., Lee T.H., Excell P.S. (2010). A Crescent-Shaped Multiband Planar Monopole Antenna for Mobile Wireless Applications. IEEE Antennas Wirel. Propag. Lett..

[32] Eskandari H., Booket M.R., Kamyab M., Veysi M. (2010). Investigations on a Class of Wideband Printed Slot Antenna. IEEE Antennas Wirel. Propag. Lett..

[33] Patre S.R., Singh S.P. (2015). CPW-fed flower-shaped patch antenna for broadband applications. Microw. Opt. Technol. Lett..

[34] Dastranj A. (2017). Very small planar broadband monopole antenna with hybrid trapezoidal-elliptical radiator. IET Microw. Antennas Propag..

[35] Boutejdar A., Abd Ellatif W. (2016). A novel compact UWB monopole antenna with enhanced bandwidth using triangular defected microstrip structure and stepped cut technique. Microw. Opt. Technol. Lett..

[36] Bozdag G., Secmen M. (2019). Compact wideband tapered-fed printed bowtie antenna with rectangular edge extension. Microw. Opt. Technol. Lett..

[37] Singhal S., Singh A.K. (2017). Asymmetrically CPW-Fed Hourglass Shaped UWB Monopole Antenna with Defected Ground Plane. Wirel. Pers. Commun..

[38] Singhal S. (2017). Asymmetrically fed octagonal Sierpinski band-notched super wideband antenna. J. Comput. Electron..

[39] Gyasi K.O., Wen G., Inserra D., Huang Y., Li J., Ampoma A.E., Zhang H. (2018). A Compact Broadband Cross-Shaped Circularly Polarized Planar Monopole Antenna with a Ground Plane Extension. IEEE Antennas Wirel. Propag. Lett..

[40] Birwal A., Singh S., Kanaujia B.K. CPW-fed broadband slot antenna for GNSS and Wifi applications. Proceedings of the 2018 IEEE Indian Conference on Antennas and Prop0gation (InCAP).

[41] Atashpanjeh E., Rezaei P. (2020). Broadband Conformal Monopole Antenna Loaded with Meandered Arms for Wireless Capsule Endoscopy. Wirel. Pers. Commun..

[42] Dang D.N., Seo C. (2019). Compact High Gain Resonant Cavity Antenna with via Hole Feed Patch and Hybrid Parasitic Ring Superstrate. IEEE Access.

[43] Sheik B.A., Sridevi P.V., Raju P.V.R. (2020). E-Shaped Patch Antennas for Multitasks/Uninterrupted 5G Communications. Wirel. Pers. Commun..

[44] Joshi A., Singhal R. (2020). Probe-Fed Hexagonal Ultra Wideband Antenna Using Flangeless SMA Connector. Wirel. Pers. Commun..

[45] Pang J., Zhu M., Yu G., Zhou H. (2020). A Nona-band narrow-frame antenna with a defected ground structure for mobile phone applications. Microw. Opt. Technol. Lett..

[46] Huang B., Li M., Lin W., Zhang J., Zhang G., Wu F. (2020). A Compact Slotted Patch Hybrid-Mode Antenna for Sub-6 GHz Communication. Int. J. Antennas Propag..

[47] Awl H.N., Abdulkarim Y.I., Deng L., Bakır M., Muhammadsharif F.F., Karaaslan M., Unal E., Luo H. (2020). Bandwidth improvement in bow-tie microstrip antennas: The effect of substrate type and design dimensions. Appl. Sci..

[48] da Silva Júnior P.F., Santana E.E.C., da Silvério Freire R.C., da Fonseca Silva P.H., Souza e Silva Neto A. (2019). Bio-inspired petal-shape UWB antenna for indoor applications. Adv. Intell. Syst. Comput..

[49] Bird T.S. (2009). Definition and Misuse of Return Loss [Report of the Transactions Editor-in-Chief]. IEEE Antennas Propag. Mag..

